# Supplementary material for: Concurrent Gene Insertion, Deletion, and Inversion during the Construction of a Novel Attenuated BoHV-1 Using CRISPR/Cas9 Genome Editing
Source: Vet Sci. 2022 Mar 30;9(4):166. doi: 10.3390/vetsci9040166 (PMC9029512; doi:10.3390/vetsci9040166)
Supplement: Supplementary file 1 [file vetsci-09-00166-s001.zip › vetsci-1596490-supplementary.pdf]

Supplementary Figure S1. Cloning and purification of BoHV-1 gE/EGFP+.

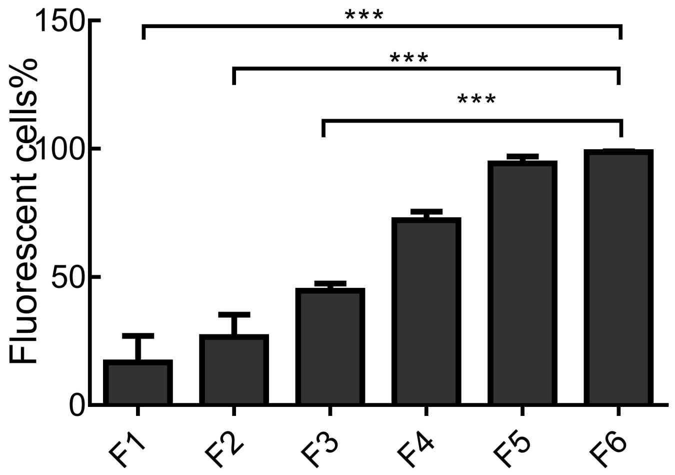

Figure S1. Purify 6 passages (F1–F6) by plaque cloning, and the percentage of fluorescent cells to total cells in each passage was calculated using Image J software. *** $p < 0.001$.
